# Maintaining energy provision in the heart: the creatine kinase system in ischaemia–reperfusion injury and chronic heart failure

**DOI:** 10.1042/CS20230616

**Published:** 2024-04-19

**Authors:** Craig A. Lygate

**Affiliations:** Division of Cardiovascular Medicine, Radcliffe Department of Medicine, University of Oxford, United Kingdom

**Keywords:** adenosine triphosphate, cardiac energetics, creatine kinase, heart failure, ischaemia-reperfusion injury

## Abstract

The non-stop provision of chemical energy is of critical importance to normal cardiac function, requiring the rapid turnover of ATP to power both relaxation and contraction. Central to this is the creatine kinase (CK) phosphagen system, which buffers local ATP levels to optimise the energy available from ATP hydrolysis, to stimulate energy production via the mitochondria and to smooth out mismatches between energy supply and demand. In this review, we discuss the changes that occur in high-energy phosphate metabolism (i.e., in ATP and phosphocreatine) during ischaemia and reperfusion, which represents an acute crisis of energy provision. Evidence is presented from preclinical models that augmentation of the CK system can reduce ischaemia–reperfusion injury and improve functional recovery. Energetic impairment is also a hallmark of chronic heart failure, in particular, down-regulation of the CK system and loss of adenine nucleotides, which may contribute to pathophysiology by limiting ATP supply. Herein, we discuss the evidence for this hypothesis based on preclinical studies and in patients using magnetic resonance spectroscopy. We conclude that the correlative evidence linking impaired energetics to cardiac dysfunction is compelling; however, causal evidence from loss-of-function models remains equivocal. Nevertheless, proof-of-principle studies suggest that augmentation of CK activity is a therapeutic target to improve cardiac function and remodelling in the failing heart. Further work is necessary to translate these findings to the clinic, in particular, a better understanding of the mechanisms by which the CK system is regulated in disease.

## Background

### The heart as an energetic organ

Skeletal muscle fatigues and will stop performing work, but cardiac muscle doesn’t have that luxury and must beat continuously, ∼3 billion times in a lifetime. Such consistency necessitates robust failsafe mechanisms that includes considerable built-in redundancy. The external energy cost to the left ventricle (i.e., stroke work) is ∼1.1 Joules per beat, which does not include potential energy or basal cellular metabolism [1]. To meet these considerable demands, the cardiomyocyte is packed with mitochondria, accounting for up to 40% of the cell volume and producing 95% of the cellular ATP requirements via oxidative phosphorylation, with the rest coming from glycolysis [2]. Remarkably, the total amount of ATP in an adult human heart is only approximately 0.6 g, sufficient to power approximately 20 beats [3], yet the heart requires 6 kg of ATP per day [4], which necessitates turnover of the entire ATP pool every 10 s [2].

This review will focus on myocardial energetics, that is, the kinetics and thermodynamics of ATP production and utilisation in the heart, which includes storage, transport and buffering. For a more generalised review of cardiac energy metabolism that includes substrate utilisation and oxidative phosphorylation, the reader is referred to the following high-quality reviews [2,4–6].

### Maintaining ATP levels in the healthy heart

ATP levels in the healthy heart do not change regardless of workload [7]. To achieve this, all vertebrates make use of the creatine kinase (CK) phosphagen system to smooth out mismatches between ATP supply and demand, buying time until ATP production can be ramped up in response to rising mitochondrial calcium levels. Mitochondrial CK (Mt-CK) forms octamers located within the mitochondrial intermembrane space and catalyses the transfer of the γ-phosphoryl group from newly generated ATP onto creatine to form phosphocreatine (PCr) and ADP, which can then stimulate further oxidative phosphorylation [4,8,9]: Creatine + ATP ↔ Phosphocreatine +ADP + H^+^

**Figure 1:**
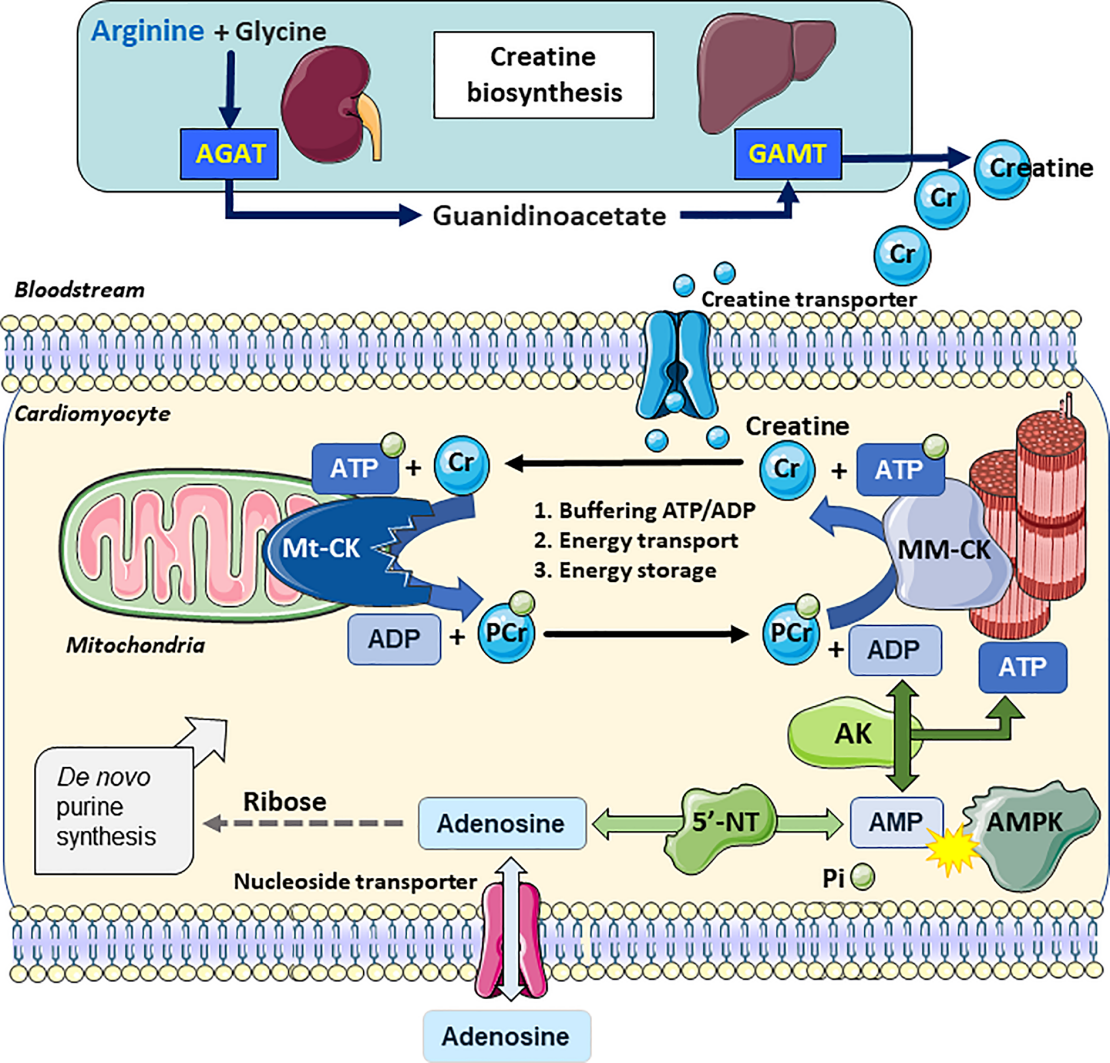
Creatine biosynthesis and myocardial phosphotransfer Creatine biosynthesis starts with arginine-glycine amidinotransferase (AGAT) located predominantly in the kidney, which combines glycine and arginine to form the creatine precursor guanidinoacetate (GA). GA is carried in the bloodstream to the liver, where it is methylated by guanidinoacetate *N*-methyltransferase (GAMT) to form creatine, which is released back into the bloodstream. Uptake into cardiomyocytes is via the specific plasma membrane creatine transporter (SLC6A8), where mitochondrial creatine kinase (Mt-CK) catalyses the transfer of a phosphoryl group from ATP to form ADP and phosphocreatine (PCr). PCr accumulates to high levels and is available for the regeneration of ATP at times of high demand catalyzed by cytosolic isoforms such as MM-CK. Liberated creatine diffuses back to mitochondria to stimulate further oxidative phosphorylation. Adenylate kinase (AK) can interconvert 2 ADP molecules to from ATP, inorganic phosphate (Pi) and AMP, which can activate AMP-activated protein kinase (AMPK). AMP is dephosphorylated by 5′-nucleotidase (5′NT) to adenosine, which is lost from the cell via an equilibrative nucleoside transporter. The pentose phosphate pathway provides ribose via multi-step reactions (dashed line), which is necessary for *de novo* protein synthesis and replenishment of the adenine nucleotide pool (ATP + ADP + AMP). Created using Servier Medical Art by Servier which is licensed under a Creative Commons Attribution 3.0 Unported License http://www.servier.com/slidekit

PCr is relatively small and is less polar, which means it is more diffusible and can accumulate to higher levels, approximately double that of ATP (e.g., ∼20 and 10 mM, respectively in healthy human heart) [3]. Thus, PCr represents a highly mobile energy reserve that allows very rapid regeneration of ATP (∼10 mM/s) catalysed by cytosolic CK isoenzymes [3], often located in close proximity or directly coupled to key ATP-consuming proteins, for example, the sarcoendoplasmic reticulum calcium ATPase (SERCA) or the myosin ATPase [10]. The free creatine liberated in this reaction acts as a signal to increase mitochondrial production of ATP [7]. The heart expresses four isoenzymes of CK, with the most abundant being muscle-type, which forms a homodimer MM-CK comprising ∼67% of total CK activity, compared with 35% for mitochondrial CK (Mt-CK). There is also a small amount of brain-type CK (∼1%), which can form homodimers (BB-CK) or heterodimers (MB-CK) [11] (Figure 1).

A key aspect to note in this paradigm is compartmentalisation, such that ATP and ADP are not required to diffuse within the cardiomyocyte. The rapid turnover by CK ensures that local reactant concentrations remain thermodynamically favourable, for example, maintaining low (ATP/ADP) ratio at the mitochondria to promote ATP production and high (ATP/ADP) at the ATPases, which maximises the Gibbs free energy of ATP hydrolysis (│Δ*G*_ATP_│), i.e. the energy per mole of ATP available to perform work [8]. This is a fundamentally important parameter that integrates all energetic process in the cell and determines the driving force for ATP utilising reactions to proceed, i.e., the difference between │Δ*G*_ATP_│for the cell and the│Δ*G*│for a given ATPase represents the energy reserve driving that reaction. Since the SERCA pump has the lowest energy reserve, it is theoretically most susceptible to impaired energetics, and this is hypothesised to be a mechanistic driver of diastolic dysfunction [12]. │Δ*G*_ATP_│is determined by the relative concentrations of ATP, ADP and inorganic phosphate (Pi), but ADP is difficult to measure since it is mostly protein bound, so must be estimated via the CK equilibrium reaction, which illustrates the dependence of ADP on the relative concentrations of free creatine to PCr [3,13].

$$\left[ADP\right]=\frac{\left[ATP\right]\left[Free\;Cr\right]}{\left[PCr\right]\left[H^{+}\right]1.66\times 10^{9}}$$

This interdependent relationship has been neatly demonstrated in healthy isolated rat hearts, where inhibition of CK, reduced both the energy reserve and the contractile reserve [12].

Adenylate kinase (AK) works in concert with CK to contribute approximately 15% of phosphotransfer in the healthy heart by catalysing the reversible reaction 2ADP ↔ ATP + AMP [14], which enables the interchange of phosphoryl groups between adenine nucleotides (i.e. AMP, ADP and ATP), the sum of which is termed the total adenine nucleotide (TAN) pool [15,16]. Cytosolic AK-1 is the predominant isoform representing the major source of intracellular AMP in the heart [17]. The remaining 5–7% of intracellular energy flux occurs via glycolysis [14].

### Where does creatine come from?

Creatine is found in animal products and a standard omnivorous diet will provide ∼50% of total body requirements (mostly found in skeletal muscle) [18]. The remainder comes from a two-step *de novo* biosynthesis, but not in cardiomyocytes, since they do not express the essential enzymes arginine:glycine amidinotransferase (AGAT; EC2.1.4.1) and guanidinoacetate *N*-methyltransferase (GAMT; EC 2.1.1.2) (see Figure 1 for schematic). Instead, AGAT is mostly expressed in the kidney, where arginine and glycine are combined to produce guanidinoacetate (GA), which is then transported in the bloodstream to the liver for methylation by GAMT to form creatine [9,18]. Cardiomyocytes therefore take-up creatine against a large concentration gradient via a specific plasma membrane creatine transporter (CrT; SLC6A8) and this is the only known way for creatine to enter the cardiomyocyte, where approximately two-thirds of intracellular creatine is maintained as phosphocreatine [19]. Loss of creatine from cardiomyocytes is slow via spontaneous cyclisation to form membrane-permeable creatinine, and in this way ∼2–3% of the total creatine pool is lost and needs to be replaced every day [20].

## Ischaemia–reperfusion injury

### What happens in the ischaemic heart?

**Figure 2:**
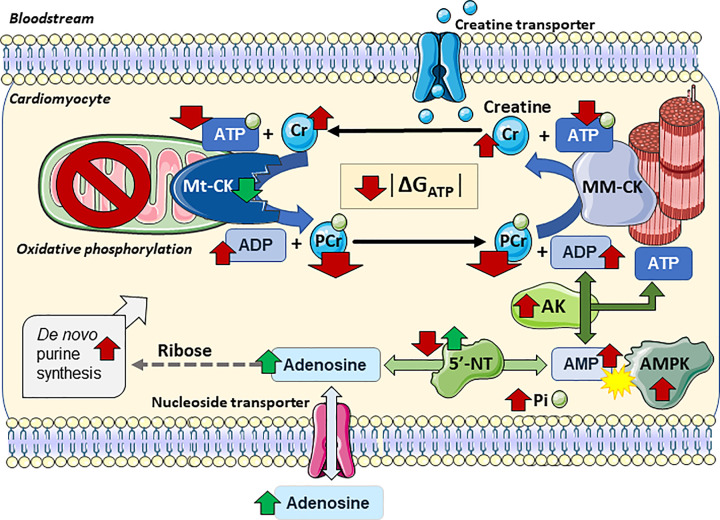
Schematic showing effect of acute ischaemia and reperfusion on myocardial high-energy phosphate metabolism Red arrows indicate directional change during ischaemia for metabolites and enzyme activities. Upon reperfusion metabolite levels gradually recover, but specific changes are shown by the green arrows. Absence of oxygen shuts down oxidative phosphorylation in the mitochondria and the cardiomyocyte becomes dependent on anaerobic glycolysis. ATP production drops dramatically, but ATP levels are temporarily buffered by rapid transfer of phosphoryl groups from PCr, ATP generation via glycolysis and adenylate kinase (AK) activity to convert 2× ADP into ATP. Thus, PCr levels drop rapidly, ATP more slowly, while free creatine, ADP, AMP and inorganic phosphate (Pi) all increase. These metabolite changes combine to drastically reduce the free energy available from ATP hydrolysis (│Δ*G*_ATP_│) and the lack of energy to power ionic homeostasis leads to Ca^2+^ accumulation. An increase in AMP relative to ATP activates AMPK to stimulate energy saving pathways and shut down anabolic ones. Low pH inhibits 5′-nucleotidase (5′NT) during ischaemia, but 5′NT is stimulated when pH recovers during reperfusion: adenosine levels rise, but with loss of adenosine across the plasma membrane. *De novo* purine synthesis is stimulated by ischaemia, but remains a bottleneck for replenishment of adenine nucleotides. Reactive oxygen species released by mitochondria during reperfusion can damage key enzymes involved in ATP production thereby impairing recovery, including Mt-CK, which is particularly susceptible. Created using Servier Medical Art by Servier which is licensed under a Creative Commons Attribution 3.0 Unported License http://www.servier.com/slidekit

Ischaemia represents a major energetic crisis for the heart since, by definition, oxidative phosphorylation can no longer take place and ATP demand massively outstrips supply (see Figure 2 for a schematic) [13]. For practical and ethical reasons, these early events are virtually impossible to study in patients, so most of our understanding is gleaned from animal models (particularly isolated perfused hearts): PCr levels drop within seconds as it is rapidly converted to ATP, but this is only sufficient to maintain full cardiac function for a matter of seconds and the heart will quickly lose all contractile force. This sudden reduction in demand means ATP levels reduce more gradually, partially buoyed by glycogen stores being used for anaerobic glycolysis. However, cardiomyocytes still require ATP for basal energy metabolism, such as ionic homeostasis, and therefore intracellular calcium rises gradually during this period and will eventually result in myocardial contracture and permanent damage [21,22].

Timely revascularisation of the ischaemic heart is standard medical practice and reduces the ischaemic damage, but heralds a second phase of cellular damage linked to reperfusion, collectively termed ischaemia–reperfusion injury (I/R). The reintroduction of oxygenated blood results in a huge burst of reactive oxygen species (ROS) as the electron transport chain reactivates in the presence of accumulated succinate. Alongside low ATP levels, rising pH and elevated Ca^2+^, this promotes opening of the mitochondrial permeability transition pore (mPTP). This transient pore acts to dissipate the mitochondrial membrane potential, thereby uncoupling respiration and resulting in necrotic cell death [23,24].

### Ischaemia and the adenine nucleotide pool

Adenylate kinase takes on particular importance during times of metabolic stress and is upregulated immediately following ischaemia [25]. It represents a mechanism to extract energy from two ADP molecules by making use of the β-phosphoryl groups to form an additional molecule of ATP plus one of AMP [15]. The AMP formed in this process plays an important signalling role by stimulating AMP-activated protein kinase (AMPK), which acts as master regulator to promote energy saving and closedown energy costly pathways [26]. Hearts from AK-1 knockout mice stopped beating more rapidly at the onset of ischaemia, and had impaired functional recovery and reduced coronary flow on reperfusion associated with reduced ATP, ADP and PCr levels [27]. One key explanation for this, is that AMP is degraded by a specific cytosolic 5′-nucleotidase (5′-NT) to form adenosine, which can cross the plasma membrane via an equilibrative nucleoside transporter [28] (Figure 2). The activity of 5′NT is inhibited by the low pH during ischaemia, but generation and release of adenosine increases upon reperfusion as pH recovers [29]. The subsequent release of adenosine acts as a local vasodilator to beneficially increase coronary blood flow and reduce injury. This is a double-edged sword, however, since it contributes to the loss of total adenine nucleotides, which has a detrimental effect on the resynthesis of ATP. This likely explains why mice overexpressing (OE) AK1 did not exhibit a clear-cut cardioprotective effect when subjected to I/R injury [30].

Although some TAN loss is counteracted by salvage pathways, the *de novo* synthesis of purines is very slow involving a multi-step process with numerous substrates and co-factors [31]. During recovery from ischaemia, *de novo* purine synthesis increases 6-fold, yet even at this rate, re-synthesis of adenine nucleotides takes days [31,32]. In animal models, supplementation with D-ribose has been shown to partially overcome this bottleneck, since endogenous availability via the pentose phosphate pathway can be a limiting factor. However, even with intravenous ribose treatment, purine synthesis rate remains too low to be beneficial in the immediate setting of acute ischaemia, for example, in a dog model of I/R, ribose treatment did not alter ATP levels after 3 h [33]. In contrast, the total creatine pool (the sum of phospho- and free creatine) is much more resilient to ischaemic injury and is maintained even after 1 h of ischaemia [34], hence PCr levels recover quickly even after prolonged periods of ischaemia.

### CK system in ischaemia–reperfusion injury

Much of what we know comes from animal models. Early studies made extensive use of pharmacological inhibition, for example, iodoacetamide has been used as a rapid and irreversible inhibitor of all CK isoenzymes [35], although as an alkylating agent it is toxic and indiscriminate [36]. Creatine analogues such as β-guanidinopropionic acid (β-GPA) can compete with creatine for cellular uptake at the CrT, but are relatively poor substrates for CK, thereby representing a model of partial creatine replacement and reduced CK activity [37]. However, creatine deficiency takes weeks, which is ample time to develop compensatory adaptations and there is always some residual creatine [38,39].

Over the past 30 years, genetically modified mouse models of loss and gain of CK function have been commonly used (summarised in Tables 1 and 2). While these represent a more precise lesion compared with pharmacological approaches, they do come with specific caveats. Most notably, all of the CK knockout and creatine-deficiency models (AGAT and GAMT KO) are constitutive, whole-body knockouts, whereby the whole-body metabolic phenotype may impact on cardiac function and a lasting effect from developmental deficiency cannot always be discounted (for detailed discussion see [40]).

**Table 1:** Genetic loss-of-function models of the creatine kinase system

Mouse model	Cardiac phenotype
**MCK-KO**	● Whole body deficiency of M-CK [171]
Ckm^tm1Bew^	● Limitations: early studies on mixed 129Sv and C57BL/6 genetic background
	● Mild energetic impairment insufficient to alter cardiac function [167,168,172,173]
**MtCK-KO**	● Whole body deficiency of mitochondrial CK [174]
Ckmt2^tm1Bew^	● Limitations: mixed 129Sv and C57BL/6 genetic background in early studies
	● Seldom studied as single KO; typically, as double KO
	● No cardiac dysfunction @ 1 year on C57BL/6 background [129]
**CK-dKO**	● Whole body deficiency of M-CK and Mt-CK (residual CK activity <4%) [175]
Ckm^tm1Bew^/Ckmt2^tm1Bew^	● Limitations: mixed 129Sv and C57BL/6 genetic background in early studies; adaptations to cytoarchitecture [132]
	● Mixed: reduced contractile reserve by echo [176]
	● Mixed: variable levels of LV hypertrophy [40]
	● Mixed: LVH in M+F @ 1 year, but congestive heart failure in males only [131]
	● Mixed: unchanged post-infarct remodelling and heart failure [153]
	● C57BL/6: no LVH and only mild LV dysfunction *in vivo* @ 1 year [131]
**GAMT-KO**	● Whole-body deficiency of creatine if fed a creatine-free diet [134]
Gamt^tm1Isb^	● Limitations: accumulation of creatine pre-cursor may be toxic and can participate in CK reaction; low fat, muscle mass, and body weight [44]
C57BL/6J	● No obvious cytoarchitecture and mitochondrial adaptations [135]
	● Reduced LV systolic pressure and blunted contractile reserve [44]
	● Increased susceptibility to ischaemia/reperfusion injury [44]
	● Reduced cardiac stroke work >1 year - energy-sparing adaptation? [136]
	● Unchanged post-MI remodelling and heart failure development [137]
	● Normal forced and voluntary running capacity [137]
**AGAT-KO**	● Whole-body deficiency of creatine if fed a creatine-free diet [138]
Gatm^tm1.1Isb^	● Limitations: homoarginine deficiency; very low fat, organ and body weights, progressive skeletal muscle atrophy (rescued by creatine) [138,139,177]
C57BL/6J	● Protected against metabolic syndrome when fed a high-fat diet [138]
	● Reduced LV systolic pressure (rescued by creatine) [20]
	● Impaired contractility, relaxation, contractile reserve (rescued by hArg) [20]
	● Intolerant of chronic hypoxia (rescued by creatine) [178]
	● Dietary creatine withdrawal post-MI reduces heart creatine, but does not affect HF development [154]
**CrT-KO**	● Whole-body creatine transporter (slc6a8) knockout [140]
B6(Cg)Slc6a8^tm1.2Clar^/J	● Limitations: very low body weight; variable levels of residual creatine in tissues, e.g., 22% in heart in initial study [140]
C57BL/6J	● Low heart weight with residual creatine 0.3%, but histologically normal. Severe skeletal muscle atrophy with early mortality by 7 months [143]
**CrT-KO**	● Whole-body creatine transporter (slc6a8) knockout [141]
Slc6a8^tm1e(KOMP)Wtsi^	● Limitations: low body weight; variable levels of residual creatine in tissues, e.g., 7% in heart, 11% in skeletal muscle, normal in kidney [141]
C57BL/6J	● Skeletal muscle atrophy with impaired strength and running distance [141]
**CrT-KO**	● Whole-body creatine transporter (slc6a8) knockout [142]
Slc6a8^−/y^	● Limitations: low body weight; variable levels of residual creatine in tissues, e.g., 1% in heart, 36% in skeletal muscle [142]
C57BL/6N	● Prolonged QTc interval; increased LV dimensions and mass when indexed to body weight (caution: may reflect extremely low body wt.); preserved systolic function; unexplained early mortality [144]

**Table 2:** Genetic gain-of-function models of the creatine kinase system

Mouse model	Cardiac phenotype
**MCK-OE**	Withdrawal of tetracycline activates cardiac-specific M-CK overexpression [166]
Tet-off system	Limitations: caution with appropriate controls for Cre expression and tetracycline off-target effects
α-MHC promoter	CK activity +70% with no clinical phenotype at baseline
Tg(Myh6-Ckm)	Protects against pressure-overload induced chronic heart failure and improves survival by maintaining myocardial energetics. Cardiac function deteriorates when transgene switched off [166]
C57BL/6	LV remodelling response is unaltered [61,166]
	Improved recovery from ischaemia/reperfusion [52]
	Protects against doxorubicin cardiotoxicity [179]
**MtCK-OE**	Cardiac-specific Mt-CK overexpression [51]
Mouse *Ckmt2*	Limitations: increase in CK activity only 27% (cf. MtCK-OE below)
α-MHC promoter	No clinical phenotype at baseline [51]
Rosa26^tm1(Myh6-Ckmt2)^	Improves functional recovery and protects against I/R injury by delaying mPTP opening [51]
C57BL/6J	Does not protect against pressure-overload induced chronic heart failure despite maintaining normal PCr/ATP [169]
**MtCK-OE**	Withdrawal of tetracycline activates cardiac-specific Mt-CK overexpression by ∼2.8-fold [61]
Tet-off system	Limitations: caution with appropriate controls for Cre expression and tetracycline off-target effects
α-MHC promoter	No clinical phenotype at baseline, but increased ATP production via CK
C57BL/6	Protects against pressure-overload induced LV remodelling and chronic heart failure due to improved energetics and reduced oxidative stress. Protective effect lost with creatine deficiency (i.e. when crossed with GAMT KO)
	LV hypertrophy due to isoproterenol is attenuated
**CrT-OE**	Constitutive cardiac-specific overexpression of creatine transporter (slc6a8) [54]
Rabbit CrT	Limitations: highly variable creatine levels with >100% increase causing LVH and heart failure [54,180]; transgene expressed during development
MLC2v promoter	Moderate creatine elevation (+20 - 100%) improves functional recovery and protects against I/R injury by improving energetics and delaying mPTP opening [55]
Tg(Myl2-Slc6a8)-55 C57BL/6J	Moderate creatine elevation does not protect against post-MI remodelling and heart failure [55,164]
**CrT-OE**	Constitutive cardiac-specific overexpression of creatine transporter (slc6a8) [181]
Human CrT	Limitations: only 4% of cardiomyocytes express transgene
α-MHC promoter	PCr/ATP elevated, but only due to lower ATP and unchanged PCr
FVB background	Mild LV hypertrophy, but normal systolic and diastolic function

#### Loss-of-function

Animal models have consistently shown that loss of CK function is detrimental to the ischaemic heart. For example, isolated rat hearts perfused with the CK inhibitor, iodoacetamide, developed higher end-diastolic pressures during hypoxia and recovery was impaired due to an inability to produce PCr [35], with similar results reported in the ischaemic rabbit heart [41]. Furthermore, Mt-CK activity reduces during ischaemia and was found to closely correlate with recovery of LV function [42]. Comparable results have been obtained in genetically modified mice. Double CK knockout mice (i.e. deficient in both M- and Mt-CK) have increased accumulation of intracellular calcium and delayed functional recovery following I/R *ex vivo* [43], with similar results obtained for creatine deficiency due to GAMT knockout, where recovery of contractile function was around half that of wild-type (WT) hearts [44].

#### Gain-of-function

The molecular composition of the mPTP remains a matter of debate [45], and while Mt-CK is not a requisite component, it does appear to influence pore formation. For example, creatine has been shown to inhibit mPTP opening in mice genetically modified to express Mt-CK in the liver and this was dependent on the localisation of Mt-CK within the mitochondrial membrane and close coupling to oxidative phosphorylation [46]. Mechanistically, this suggests a protective effect related to enzymatic activity, but a potential mechanical role has also been proposed, since octameric Mt-CK precisely spans the mitochondrial inter-membrane space creating contact points that provide structural stability and contribute to membrane integrity [47,48].

Transient overexpression of Mt-CK in immortalised cardiomyocyte-like cell lines (e.g., mouse HL-1 and human AC16) improved cell survival following hypoxia/reoxygenation [49,50] and corresponding cardioprotection has since been reported in a mouse model. Mice with cardiomyocyte-specific overexpression of Mt-CK had a modest 27% increase in Mt-CK activity, which did not alter baseline cardiac metabolism or function, but significantly reduced ischaemic contracture and improved functional recovery upon reperfusion *ex vivo*. A reduction in myocardial infarct size from 55% to 29% was observed *in vivo* and isolated cardiomyocytes from Mt-CK hearts delayed opening of the mPTP, although notably, an effect on mitochondrial membrane stability was not detected [51]. M-CK overexpression in mouse heart is also cardioprotective in an *ex vivo* I/R protocol, with contractile function recovering to 65% of baseline compared with only 14% in WT hearts and accompanied by more rapid recovery of PCr levels [52].

Dietary creatine supplementation is ineffectual at increasing myocardial creatine levels due to compensatory down-regulation of the CrT [53]. However, this can be circumvented by cardiac-specific overexpression of the CrT, which resulted in mice with highly variable levels of myocardial creatine for reasons that remain unclear. Mice with total creatine 2-fold above normal eventually developed left ventricular hypertrophy (LVH) and cardiac dysfunction, however, mice with more moderate elevation (1.2–2-fold) are functionally normal and have elevated levels of myocardial glycogen stores [54–56]. When these moderate mice were subjected to I/R *in vivo*, the infarct size was 27% smaller than WT and negatively correlated with tissue creatine levels. Langendorff-perfused CrT-OE hearts had higher PCr and │Δ*G*_ATP_│at baseline, which fell more slowly during ischaemia and rapidly returned to supra-normal levels upon reperfusion. This was associated with significantly improved functional recovery and blunting of post-ischaemic hypercontractility, suggesting a reduction in Ca^2+^ accumulation and rapid return to ionic homeostasis due to an augmented energetic reserve. This cardioprotective effect of CrT overexpression appears to be robust, having been recapitulated in both sexes, in the presence of pre-existing LVH, and was additive to cardioprotection by cold cardioplegia solution [57].

It has been suggested that creatine may protect due to direct antioxidant activity, for example, scavenging superoxide anions and peroxynitrite [58,59]; however, direct evidence for a significant antioxidant activity in the intact heart is not strong. HL-1 cells loaded with creatine have been shown to delay opening of the mPTP in response to ROS, but this was not due to a direct antioxidant effect since the same cells exposed to H_2_O_2_ could not attenuate the signal from an oxidation-sensitive fluorescent dye [55]. Furthermore, when isolated mouse hearts were perfused with hydrogen peroxide or doxorubicin, the resultant dysfunction was not ameliorated or exacerbated by the presence or absence of myocardial creatine [60].

A direct link between ROS formation and Mt-CK is more obvious, since Mt-CK regulates mitochondrial ADP levels, and ADP acts to stimulate respiration, which is the largest source of cellular ROS [5]. While creatine is required for this reaction, it is the active coupling of Mt-CK to inner membrane components that appears to be critical for protection [47]. Other mechanisms may be at play, since isolated cardiomyocytes from MtCK-OE mice were found to be more resistant to H_2_O_2_ challenge and this was associated with increased expression of antioxidant proteins [61].

In summary, there is broad agreement in the literature that an intact CK system is critically important in the acute response and recovery from myocardial ischaemia. Furthermore, that augmenting either creatine levels (via the CrT) or CK activity can be cardioprotective, preventing cell death and improving recovery of contractile function. However, there are currently no tools available to achieve this pharmacologically, hence further research is required to understand the mechanisms that underpin the regulation of these therapeutic targets *in vivo*.

### What is known about regulation of CK and the CrT?

Creatine is taken-up into cardiomyocytes via a specific transporter which is sodium and chloride dependent, so any factors that influence the Na^+^ gradient could regulate creatine uptake [62]. Creatine itself provides a real-time regulatory feedback mechanism, strongly inhibiting further uptake when stores are high and stimulating CrT activity by up to 7-fold when stores are depleted, predominately via non-transcriptional mechanisms [63]. Thioredoxin-interacting protein (*Txnip*) may play a role in this response, since it is expressed when creatine levels are high and gene silencing increases creatine accumulation *in vitro* [64]. A number of other potential regulators have been identified, but mostly in non-cardiac cell lines or in artificial expression systems, so the relevance to myocardial biology is mostly unknown (summarised in [19]). Several of these candidates converge on the AMPK signalling pathway, including AMPK itself, mTOR and PGC1-α via ERRα, which is physiologically plausible as a response to increase energy generation. Notably, ERRα deficient mice exhibit a trend for reduced MtCK gene expression that is exacerbated with heart failure, depletion of myocardial PCr at high workloads, and impaired recovery of PCr following ischaemia, suggesting a potential role as a master regulator of cardiac energetics. It is postulated that the reduction in ERRα/PGC1α observed with hypertrophy could drive the reduction in CK in the failing heart [65]. Other regulators may include hormones, such as growth hormone, which increased CrT expression in a rat MI model [66]. The CrT also contains phosphorylation sites that modify creatine uptake, at least in skeletal muscle, and N-glycosylation sites that may be involved in trafficking to the cell membrane *in vitro* [19]. However, the significance of these in heart disease is unknown, partly hindered by the lack of good quality specific CrT antibodies [67].

The activity of Mt-CK will depend, in part, on the interactions with proteins it is in complex with at the mitochondrial membranes (e.g., ANT and VDAC), since this will affect the efficiency of substrate channelling, whereby intermediates are transferred directly between linked enzymes [68]. Mt-CK may also be subject to degradation by ubiquitination pathways and a number of sites for post-translational modification have been identified, e.g., phosphorylation and acetylation [68], which can protect against hypoxia/reoxygenation *in vitro* [50]. M-CK is a target for phosphorylation by protein kinase C (PKC), which regulates activity, since dephosphorylation caused a 2-fold increase in ATP generation, which may have relevance to the diabetic heart [69]. All of the CK isoenzymes are susceptible to damage and inactivation by free radicals and reactive oxygen species (ROS) [47]. Mt-CK is the most susceptible CK isoenzyme due to the mitochondrial location, where ROS can lead to dissociation from octamer to the less active dimer, although coupling of creatine at the active site can prevent this [47]. For example, peroxynitrite released during ischaemia has been shown to reduce Mt-CK activity in rat heart associated with a reduction in the octamer/dimer ratio [70].

## Cardiac energetics in chronic heart failure

**Figure 3:**
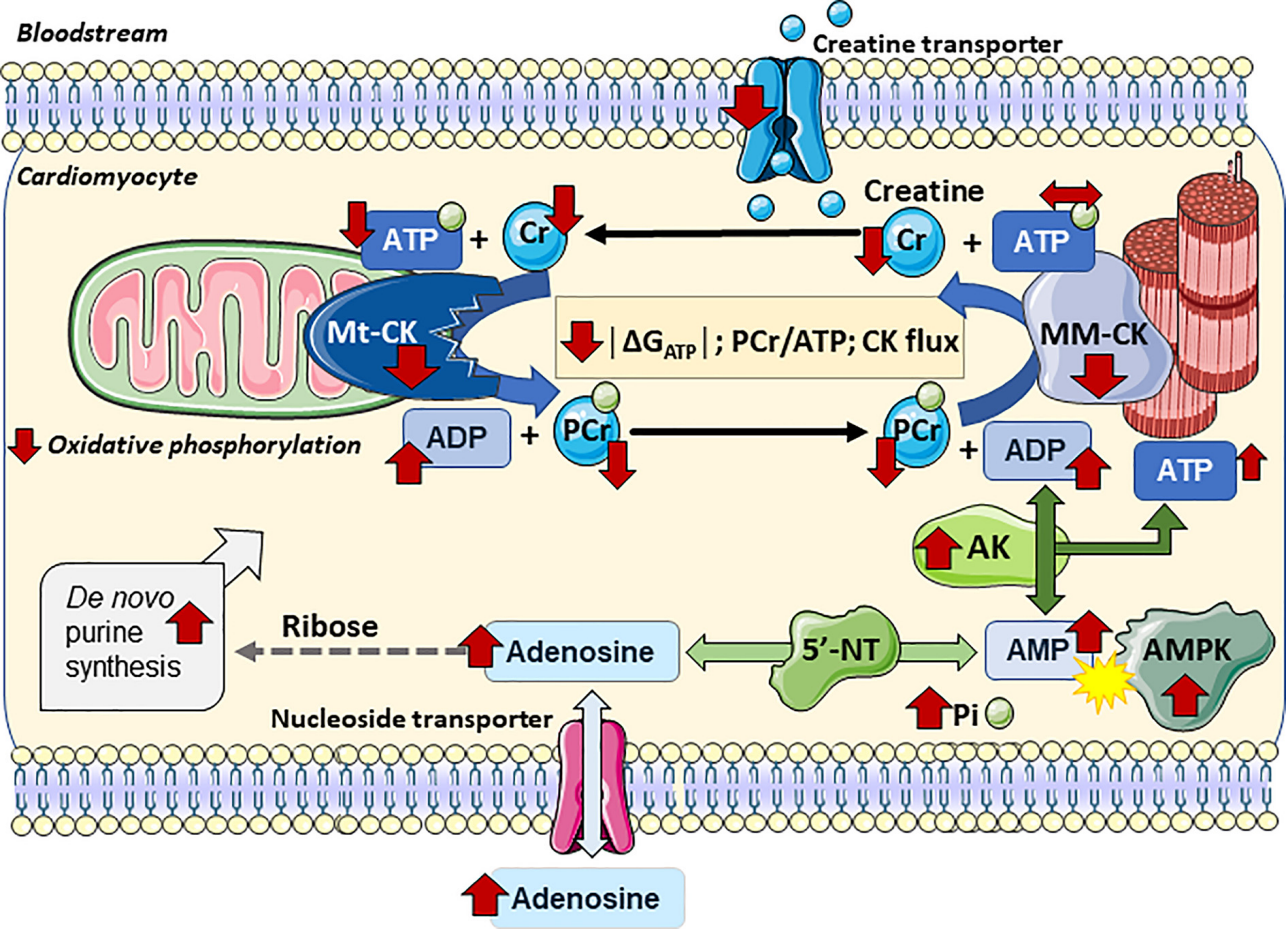
Schematic showing effect of chronic heart failure on myocardial high-energy phosphate metabolism Red arrows indicate directional change for metabolites and enzyme activities in the setting of chronic heart failure. Total creatine levels are lower due to down-regulation of the creatine transporter to reduce cellular uptake. Activity of both Mt-CK and MM-CK are substantially reduced, the former likely due to oxidative damage and the later has been linked to post-translational modification by acetylation. The result is a rise in ADP and inorganic phosphate (Pi) and a fall in CK flux, PCr/ATP ratio, and │Δ*G*_ATP_│. Absolute values of ATP are maintained for a long time due to preferential repletion by PCr, but eventually diminish in end-stage disease, which also reflects reduced ATP production via oxidative phosphorylation, attributable to multiple causes, e.g., oxidative damage, loss of control via ADP and creatine, uncoupling of the electron transport chain and changes in substrate utilisation. The relative contribution of AK to phosphotransfer is increased, which leads to accumulation of AMP and activation of AMPK. The increase in AMP provides more substrate for 5′NT leading to increased formation of adenosine and accelerated loss of total adenine nucleotides. Although this stimulates de novo purine synthesis, it is insufficient to compensate in the long term. Created using Servier Medical Art by Servier which is licensed under a Creative Commons Attribution 3.0 Unported License http://www.servier.com/slidekit

The theory that impaired energetics is a defining property of heart failure dates back to the 1930s, when low levels of creatine and phosphate were observed in failing hearts post-mortem [71]. Since then myriad studies from a wide variety of species have shown that both creatine levels and creatine kinase activity are significantly reduced, independent of the aetiology of heart failure (see Figure 3 for a schematic) [72]. For example, total creatine was reduced by 26% and CK activity by 48% in a pig model of pressure-overload [73] and by 39% and 25% respectively in a dog pacing model of heart failure, where they normalised upon recovery [74]. In the dog model, loss of both creatine and ATP was progressive, correlating with the duration of pacing, although the rate of creatine depletion was 7-fold higher [75]. It has been postulated that the decline in creatine is compensatory by keeping ADP levels from rising and thereby maintaining ATP/ADP, which in turn helps preserve ΔG_ATP_ [76] and slows the loss of adenine nucleotides [75]. The reduction in CK activity is partially compensated by alternative phospho-transfer pathways, such as AK. In the dog pacing model of heart failure, the contribution of AK increases to 21% of ATP turnover [16], and in mouse models of MI and pressure-overload, the contribution of AK and glycolytic pathways relative to CK also increased [77].

The decline in creatine levels observed in the failing heart has been attributed to a reduction in creatine uptake rather than changes in cellular efflux [62], with CrT protein expression down-regulated in both human and rat heart failure [78]. In the dog pacing model of heart failure, M-CK and MtCK were both down-regulated in terms of protein expression and activity; however, upon recovery (pacing cessation) CK RNA and protein expression did not change, but CK activity returned to normal and correlated with improved systolic function, indicating reversible post-translational regulation of CK [74]. A prime candidate for such regulation is an increase in M-CK acetylation, which was observed in samples from both human and mouse heart failure, and was inversely correlated with protein activity. Hyperacetylation at lysine residues is thought to interfere with dimer formation, and crucially, this could be reversed in *vitro* by deacetylation with Sirtuin 2 to rescue M-CK activity levels. This suggests acetylation/deacetylation pathways as potential targets to rescue M-CK activity, although notably, Mt-CK activity was unaffected [79]. In contrast, in human end-stage heart failure, CK activity increased after unloading with a left ventricular assist device commensurate with increased protein expression of CK [80], so it seems likely that regulation occurs at multiple levels. However, relatively little is known about the precise mechanisms mediating these changes, particularly in disease. A greater understanding of the mechanisms governing CK and CrT regulation would help join-the-dots between heart failure and altered energetics and inform the debate on causation. It is also necessary to identify potential molecular targets that may form the basis for therapeutic modulation.

### Non-invasive assessment of cardiac energetics in humans

The hypothesis that the failing heart is ‘energy starved’ and acts as a pathological driver of disease [4,10], is supported by a wealth of correlative data from human and animal models, although robust examples of cause and effect remain elusive. The following section will discuss the evidence available from the study of human heart failure, but first an explanation of how cardiac energetics are assessed non-invasively.

**Figure 4:**
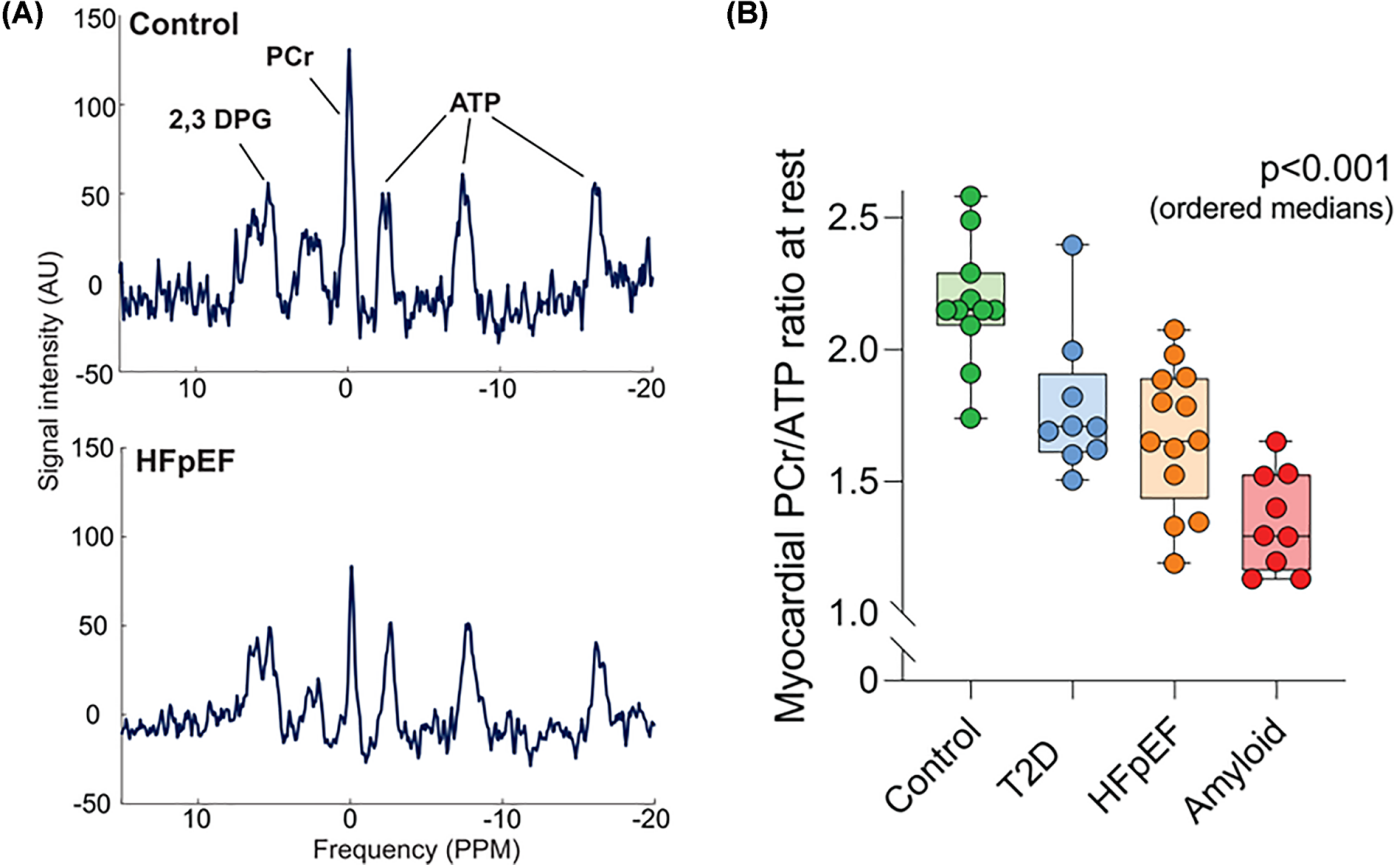
Cardiac energetics assessed by phosphorus magnetic resonance spectroscopy Panel A shows representative spectra from a healthy human control (upper panel) and a patient with heart failure with preserved ejection fraction (HFpEF). Note the large phosphocreatine (PCr) peak in the control relative to HFpEF, while the three phosphoryl peaks of ATP are relatively preserved. 2,3-DPG indicates the blood component diphosphoglycerate. Panel B shows PCr/ATP ratio across the spectrum of diastolic dysfunction from normal, type-2 diabetes (T2D), HFpEF and patients with amyloidosis. Figure adapted and reproduced with permission from Burrage *et al*. (2021) Circulation. 144, 1664–1678, https://doi.org/doi:10.1161/CIRCULATIONAHA.121.054858

Magnetic resonance spectroscopy is a close relation of MRI, but instead of visualising protons, specialist hardware allows the assessment of phosphorus levels (^31^P-MRS), thereby bestowing the ability to measure PCr and ATP in the intact heart alongside inorganic phosphate and, indirectly, pH (example spectra are shown in Figure 4). Early studies, made use of explanted or perfused animal hearts, but the development of surface coils made open-chest preparations and non-invasive studies feasible (for historical reviews see [81,82]). The first non-invasive ^31^P-MRS measurements in humans was reported in the mid-1980s [83] and the technique has since revolutionised our understanding of cardiac energetics in chronic heart failure (CHF).

The most straightforward parameter to extract from ^31^P-MRS is the ratio of PCr/ATP, since this has no units and does not require absolute quantification of metabolite concentrations, which is technically challenging [82]. The CK reaction equilibrium favours ATP synthesis by ∼100-fold, which ensures that ATP levels do not change under normal conditions, so PCr/ATP mostly reflects PCr levels, and therefore, energetic status [10]. Problems with interpretation can arise in the failing heart, since ATP levels fall slowly but progressively, declining by up to 30% in end-stage heart failure [84], driven by the loss of adenine nucleotides [75]. This may result in underestimation of PCr/ATP (i.e., pseudo-normalisation) in patients with the most severe disease, hence absolute quantification of PCr and ATP has been developed by comparison with an external reference standard. Such quantification is sensitive to a variety of acquisition parameters, but has been validated against chemical analysis of biopsy samples [82].

However, metabolite pools only tell part of the story and ignore the fact they are constantly being recycled. The rate at which this recycling happens, represents the rate of energy production within the myocardium, which, because CK operates near equilibrium, can change without necessarily altering metabolite levels [85]. This turnover can be estimated using magnetisation saturation transfer techniques, which measure the rate constant for the forward CK reaction (*k_f_*), i.e., the rate at which PCr is converted to ATP, and when this is multiplied by the concentration of PCr, we obtain CK flux [82,86]. This represents an average value for all detectable CK reactions within the volume of interest and does not inform about compartmentalisation within cardiomyocytes. Nevertheless, CK flux is a sensitive parameter with a wide dynamic range, since it combines both metabolite concentrations and enzyme activity and can therefore be more informative about energetic deficits in the heart [85]. For example, CK flux could differentiate between patients with LVH and patients with LVH and chronic heart failure, whereas PCr/ATP was reduced in both groups, thus demonstrating the importance of ATP turnover compared with metabolite concentrations [87].

Double saturation transfer techniques have also been developed to measure ATP flux (i.e. rate of ATP hydrolysis) [88,89]; however, this is inherently difficult *in vivo* due to the need to measure inorganic phosphate which overlaps with 2,3-diphosphoglycerate present in blood, and few studies have reported on this parameter [90].

### Proton spectroscopy for measurement of creatine levels

Proton spectroscopy (^1^H-MRS) has been used in a handful of studies to non-invasively measure total creatine levels in the heart. This has confirmed that creatine levels are reduced in human non-ischaemic heart failure and correlate positively with ejection fraction and negatively with circulating BNP [91]. However, the potential of ^1^H-MRS is much greater than this, since in combination with ^31^P-MRS it is possible to determine all the parameters required to non-invasively calculate Δ*G*_ATP_ *in vivo* [92]. This is technically very challenging and seldom implemented, but a key example is the study by Hirsch et al. in patients with non-ischaemic cardiomyopathy, where they demonstrate that acute infusion of allopurinol improves not just PCr/ATP, CK flux, but also │Δ*G*_ATP_│. The authors estimate that this accounts for a not insignificant energy saving of 3.8 kJ per gram of myocardium per day [93]. However, the significance of these changes to acute mechanical function were not reported.

### Energetics in human heart failure

PCr/ATP ratio in the healthy human heart is typically around 2.0, although the exact value is dependent on the sequence used, making it difficult to compare values between centres [94]. Numerous studies have shown that PCr/ATP is reduced in the failing heart (recently reviewed in [91]), with early examples focused on patients with non-ischaemic dilated cardiomyopathy (DCM) for practical reasons. Neubauer et al., demonstrated a significant correlation between PCr/ATP and markers of disease severity, for example, New York Heart Association class, ejection fraction and wall thickness, with PCr/ATP improving in response to successful drug treatment [95,96]. Furthermore, in a 2.5-year follow-up of DCM patients, PCr/ATP was found to be a better prognostic marker of cardiovascular mortality than ejection fraction, although group sizes were small (*n*=19–20) and it would be interesting to revisit the prognostic potential in larger cohorts [97]. This is a common issue for many ^31^P-MRS studies since the technique remains experimental and expensive to perform. In patients with mild-to-moderate heart failure with reduced ejection fraction (HFrEF), CK flux was found to be reduced by 50%, making it the most prominent parameter of energetic deficit, and arguably large enough to become rate limiting and thereby contribute to disease progression [98]. Keceli et al. correlated energetic parameters with LV structural remodelling in patients with HFrEF and found that hypertrophy and dilatation were associated with a reduction in CK flux and ATP levels [61]. An earlier study using the same patient data also described a correlation between CK flux and parameters of cardiac work and efficiency [99]. In a prospective study, 58 patients with stable non-ischaemic cardiomyopathy were given a ^31^P-MRS examination and followed up for a median 4.7 years. CK flux, but not PCr/ATP ratio or absolute metabolite levels, was an independent predictor of all cause and cardiovascular mortality [100].

Energetic deficits have also been described in heart failure with preserved ejection fraction (HFpEF). One such study found a step-wise reduction in PCr/ATP in healthy controls and in patients with increasing severity of diastolic dysfunction (consisting of Type 2 diabetes, HFpEF, and amyloidosis) (Figure 4). PCr/ATP correlated with the diastolic parameter E/e′ and was associated with increasing levels of exercise-induce lung congestion [101]. A separate study also showed reduced PCr/ATP in non-diabetic HFpEF patients, but did not find a correlation with diastolic dysfunction or VO_2_ max, which were instead associated with elevated myocardial triglycerides [102]. Other HFpEF pathologies also exhibit impaired energetics, for example, one-year after an acute takotsubo episode, patients had normal ejection fraction, but exhibited reduced LV strain indices and limited exercise capacity, which was associated with reduced PCr/ATP [103].

Hypertrophic cardiomyopathy (HCM) represents a key example where the precise genetic mutations and molecular deficits are often known and have been linked directly to inefficiencies in contraction that increase the energy cost [104]. Patients with sarcomeric mutations had low resting PCr/ATP, which reduced further during exercise and correlated with diastolic dysfunction [105]. In a study of patients with a different sarcomeric mutation, PCr levels and CK rate constant were both significantly reduced, but did not correlate with indices of contractile function. The authors’ interpretation is that energetic changes are a consequence of the underlying mutation rather than secondary to cardiac dysfunction [106].

Most studies have been performed in highly selective, homogenous, patient groups, but energetic changes also occur in many morbidities that are associated with increased risk of developing heart failure. For example, PCr/ATP is reduced in LVH [87], atrial fibrillation [107], aortic stenosis [108], obesity [109,110] and in Type 2 diabetes [111,112]. In one notable preclinical study, it was found that diabetic mice were relatively protected from heart failure induced by pressure overload and this was attributed to restoring flexibility in energy substrate utilisation [113]. This was despite diabetic mice starting with a low PCr/ATP, and by the end of the study PCr/ATP had improved even though contractile function was worse, indicating a disconnect between energetics and function [114]. It is also becoming apparent that there is an interplay between CK reaction rate and PCr/ATP, for example, increased ATP delivery rate compensates for low PCr/ATP in obesity at rest, but does not increase further with exercise potentially limiting capacity. Notably, dietary weight loss reversed these energetic changes [109]. These findings raise questions regarding the utility of ^31^P-MRS for routine clinical use, where it will be applied to a much more heterogenous population with multiple overlapping co-morbidities. Machine learning may be one way forward, particularly in combination with comprehensive examinations that measure PCr and ATP concentrations, *kf*, and CK flux. In a recent study, data from 178 patients was used to train a neural network, which demonstrated 84% accuracy in diagnosing disease classification (healthy, DCM, HCM, or anterior MI) from these parameters alone [115]. Obviously, there are quicker and easier ways to distinguish between heart failure aetiologies, but it demonstrates proof-of-principle for a machine learning approach.

### What do changes in energetics actually represent?

When changes in energetic parameters are observed, it is worth considering what this represents at a mechanistic level, i.e., what is the link between the underlying disease state and altered energetics? The strongest relationships probably exist to link PCr/ATP and CK flux to cardiac work (or markers thereof), which makes sense, since work is a direct measure of energy expenditure. This relationship has been observed in healthy volunteers [116,117], for example, in healthy women PCr/ATP was found to decline with age and correlated with peak cardiac power output during exercise [117]. However, this is not a universal finding [118], which may, in part, reflect the difficulty of increasing workload via exercise in the magnet, but also the spare capacity that exists in the healthy heart, where CK flux is 10 times higher than ATP synthesis via oxidative phosphorylation, hence no change in CK flux is observed at high workloads [84,98].

However, this spare capacity is eroded in the disease state and cardiac energetics therefore become more strongly linked to workload. For example, in HFrEF patients, CK flux correlated with cardiac work and myocardial efficiency [99] and energetic parameters are impaired in severe aortic stenosis [108] and in hypertensive patients [119], where cardiac work is increased in order to pump against elevated afterload. Ultimately, we would expect PCr/ATP to reflect mitochondrial ATP production since 95% of energy production in cardiomyocytes is via oxidative phosphorylation (OxPhos) and 85% of this is delivered via the CK shuttle [14]. This direct link is supported by *in silico* modelling indicating that all OxPhos complexes contribute to PCr/ATP ratio under normal conditions, and that hypoxia has the single most pronounced effect on PCr/ATP ratio by limiting energy production via OxPhos [120]. There is experimental evidence to support this, for example, patients with aortic stenosis have lower myocardial oxygenation and perfusion reserve, which was associated with reduced PCr/ATP, with all parameters improving after valve replacement [121]. Similarly, Type 2 diabetics had resting PCr/ATP that was 17% lower than healthy controls and reduced by a further 12% during exercise, correlating with myocardial perfusion and oxygenation, suggesting that microvascular dysfunction is causing hypoxia which impacts on energetics [111]. A similar effect was observed in patients with flow-limiting coronary artery disease, where PCr/ATP was found to be low at baseline and reduced further with exercise, unlike healthy controls or patients with non-ischaemic heart failure. A small sub-set (*n*=5) were retested after revascularisation and demonstrated improvements in PCr/ATP that did not reduce with exercise, collectively suggesting that restriction of oxygen supply in coronary artery disease is sufficient to limit energy supply [118]. Microvascular dysfunction is likely to limit oxygen supply at higher workloads in HFpEF patients, where the ability to perform external work is blunted [122]. In contrast, hypoxia does not appear to be a feature of stable non-ischaemic HFrEF. Myocardial oxygenation in DCM patients was found to be unchanged at rest or during a stress test, furthermore, 4 h of oxygen therapy did not alter any of the energetic or functional parameters [123].

PCr/ATP is even lower in CHF patients with iron deficiency despite comparable LV dysfunction, which may reflect impaired mitochondrial function [124]. A similar argument is put forward for the effect of glyceryl trinitite infusion in healthy volunteers, where CK flux increased despite a lower workload, which the authors suggest is due to inhibition of OxPhos by nitric oxide, i.e. mitochondrial uncoupling breaks the relationship between CK and work [116]. However, one of the few studies to directly test this relationship collected myocardial samples from patients during open heart surgery but found that PCr/ATP did not correlate with mitochondrial respiration measured in permeabilised myocardial fibres. This suggests that PCr/ATP does not primarily reflect mitochondrial function and that other factors may play a role [125]. However, a major caveat is that mitochondria were isolated from right atrial appendage rather than left ventricle and may be experiencing very different cellular milieu and workloads. It should be noted that mitochondrial damage and impaired respiration is a commonly described feature of the chronically failing heart [126,127], for example, mice with pressure overload exhibit a reduction in mitochondrial energy production that is concomitant with reduced PCr/ATP and precedes development of systolic dysfunction [128].

### Do changes in cardiac energetics directly contribute to pathophysiology?

An argument often put forward is one of temporal association, i.e. where energetic changes occur early in the disease process and therefore precede and possibly contribute to later dysfunction. For example, in patients with moderate aortic stenosis, PCr/ATP is reduced before systolic dysfunction, which may therefore contribute to development of systolic failure in severe aortic stenosis when energy demands are highest [108]. However, the counterargument can also be made, i.e., that energetic deficits can exist in the absence of contractile dysfunction, which suggests they are not sufficiently severe to be a limiting factor.

Loss-of-function studies can often be informative in these debates (summarised in Table 1). If the CK system has a central role in heart failure pathophysiology, then we would expect knockout models to recapitulate this, either by spontaneous development of cardiac dysfunction or increased susceptibility to induced heart failure. Loss of M-CK on its own does not cause cardiac dysfunction even at high workloads, whereas LV hypertrophy was reported in some, but not all, studies of Mt-CK-KO and double CK-KO mice due to a mixed genetic background [40]. When backcrossed on to a C57BL/6J background, Mt-CK-KO mice no longer exhibit LVH and have normal cardiac function under dobutamine stress-test until at least 1 year of age [129]. A similar picture emerges for the CK-dKO mice, whereby male mice on a mixed genetic background had LVH and subtle changes in perfusion [130], which in our laboratory eventually developed into congestive heart failure at >1 year of age, but not on a C57BL/6J background which only exhibited mild functional impairment [131]. Thus, it appears that a relatively drastic loss of 98% of total CK activity is capable of causing a heart failure phenotype, but only in ageing males on a permissive genetic background. One potential explanation is that these are constitutive knockout models, which means compensatory adaptations may occur during development to maintain cardiac function in younger mice that are insufficient in old age. For example, redistribution of mitochondria has been described in CK-dKO hearts, which may reduce the diffusion distance for ATP [132], along with increased phosphotransfer via adenylate kinase and glycolytic pathways [133].

GAMT-KO mice have a whole-body creatine deficiency resulting in low body fat and muscle mass [134]. They don’t develop LVH and mitochondrial organisation is unchanged [135], but they have reduced LV systolic pressure and contractile reserve [44]. This becomes more pronounced at >1-year of age, when functional parameters and heart rate are also lower at rest, but in the absence of heart failure (e.g., end-diastolic pressures are normal and there is no pulmonary congestion) [136]. This reduction in function represents an estimated 33% saving in cardiac work and is concomitant with a reduction in mitochondrial cell density, but not in mitochondrial function. The authors speculate that reducing cardiac work may be an acceptable compromise in an ageing mouse where there are no survival pressures. This was borne out by the reversal of functional defects after only 10 days of dietary creatine [136]. It should be noted that GAMT-KO mice accumulate the creatine-precursor, guanidinoacetate, which may partially compensate via participation in the CK reaction, but may also accumulate to toxic levels [137], and it has not been possible to entirely disambiguate these effects.

In theory, AGAT-KO mice fed a creatine-free diet represent a cleaner model of absolute whole-body creatine deficiency [138]. However, the AGAT enzyme also synthesises the non-proteinergic amino acid, L-homoarginine, which contributes significantly to the cardiac phenotype. This consists of lower LV systolic pressure and reduced haemodynamic indices of contractility and relaxation at rest and under dobutamine stress, but in the absence of LVH. Surprisingly, dietary creatine only rescued LV systolic pressure, whereas all other parameters were rescued by dietary homoarginine, suggesting only a minor role for creatine-deficiency in driving the cardiac phenotype [20]. It should be noted that AGAT KO have extremely low body weights, with skeletal muscle atrophy and changes in body composition, which are all rescued by dietary creatine [139]. Major organ weights are also low and most likely reflect lower water content since creatine is an important cellular osmolyte [20]. These whole-body effects suggest caution in interpretation of cardiac findings, however, since creatine is synthesised in kidney and liver, a cardiac-specific deletion of either AGAT or GAMT would not be informative.

To date, there are three distinct CrT-KO mouse models reported in the literature [140–142]. One of these describes low heart weight, but with normal histology, despite residual creatine of 0.3% [143]. All three report variable levels of residual creatine in various tissues, which presumably reflects either local creatine biosynthesis or alternative mechanisms of creatine transport, however there is little evidence for either occurring in the heart [19], where creatine uptake was undetectable in the CrT-KO [143]. Only a single study reports on cardiac function, however, as with the other creatine-deficiency models, body weight is extremely low and caution is required when interpreting results normalised to body weight [144].

In-born errors of human metabolism can provide natural loss-of-function models, and in the last 30 years, creatine deficiency syndrome (CDS) has been described in patients with AGAT, GAMT and CrT mutations. CDS is typically identified early in life manifesting as mental retardation and developmental delay [145]. In addition, GAMT mutations are associated with severe epilepsy and movement disorders, most likely a toxic consequence of guanidinoacetate accumulation [146]. AGAT and GAMT mutations are rare (around 140 reported worldwide), whereas mutations in CrT may explain ∼1–2% of male children presenting with intellectual delay [145]. However, reports have understandably focussed on the neurological symptoms with very little information available on cardiac manifestations. A single case study in a 12 year old with untreated AGAT mutation described normal ECG and echocardiogram [147], while an 18-month-old boy with a CrT mutation exhibited ventricular ectopic beats, but a normal echocardiogram [148]. Two case studies have reported on genetic CK deficiency, the first describing a middle-aged man who developed ECG evidence of ischaemia during exercise in the absence of obvious coronary artery disease [149] and the second a middle-aged woman who died 11 days after an acute myocardial infarction [150]. The lack of systematic studies of cardiac function in such patients feels like a missed opportunity, but reflects the rarity of disease and ethical constraints when the population is predominately children with intellectual disability.

### Does an impaired CK system exacerbate heart failure?

The evidence presented above for a primary defect in the CK system directly causing overt dysfunction leading to a heart failure phenotype is equivocal at best. This may simply reflect compensatory adaptations and redundancy in energetic networks, which may only become apparent in the presence of widespread dysregulation, so a more nuanced question is whether an impaired CK system results in poorer outcomes in models of heart failure?

Several preclinical studies have examined CK system deficiency in the development of chronic heart failure following surgically induced myocardial infarction (MI). In studies of this type, experimental groups are typically matched retrospectively for infarct size, so that the focus is on ventricular remodelling and heart failure development, rather than response to acute injury [151]. Horn et al. fed rats the creatine analogue, β-GPA, immediately after MI with function and energetics measured *ex vivo* 8 weeks later. Mechanical function was depressed by ∼33% in both β-GPA-fed sham-operated rats and in rats post-MI, but was not made any worse in the MI + β-GPA group. This is despite the energetic defects being additive, with the MI + β-GPA hearts exhibiting an 87% reduction in PCr and 94% lower CK flux [152]. Very similar results have been obtained in the GAMT-KO mouse, where the total absence of creatine impaired contractile reserve, but 6 weeks after MI, these mice did not differ from WT controls in terms of survival, LV remodelling or mechanical function and the compensatory adaptations described in CK-KO could not be found [137]. A similar pattern emerges in two studies of CK deficiency using CK-dKO mice. These mice were on the mixed genetic background and had pre-existing LVH, dilatation, perfusion defects, however 4 weeks after MI they were indistinguishable from WT controls in terms of LV remodelling and *in vivo* function [130,153].

One argument is that constitutive knockout mouse models allow ample time for compensatory adaptations to develop, which may mask the real phenotype. To address this possibility, we fed AGAT-KO mice dietary creatine such that myocardial creatine levels were normal throughout development. They were then subjected to surgical MI and randomised 1 week later to either continuation or withdrawal of dietary creatine. After 6 weeks, creatine levels were 64% lower in the withdrawal group, but there were no differences in survival, LV remodelling or function, even under maximal stimulation with dobutamine [154]. Taken with the four studies above, this is the strongest evidence to date that an impaired CK system does not exacerbate the development of heart failure and questions whether primary deficits in cardiac energetics can be truly causal in nature.

Of course, it is possible this simply represents a species difference between rodents and humans and there are several key differences in relation to the CK system. For example, the abundance of CK isoenzymes is slightly different, with relatively more M-CK reported in human heart [11]. Total creatine levels in mice are approximately half that found in normal human hearts and mice maintain a higher fraction as PCr and a lower ADP resulting in more energy per mole of ATP [155]. The reduction in creatine levels with heart failure has been reported at 11–16%, compared with 33% in humans, and for TAN pool it is a modest 8–14% in mice, but closer to 50% loss in human heart failure [156]. However, this may simply reflect the use of early humane end-points in modern preclinical studies compared with samples from end-stage heart failure in humans, since loss of these metabolites is known to be gradual. There are many other considerations in relation to allometric scaling, most obviously in size, metabolic rate and heart rate, and yet the basal energy consumption/body atom/heart beat is apparently unchanged across species suggesting fundamental similarities [157]. Furthermore, the changes in CK system that occur with heart failure correlate with measures of cardiac function in both species, again suggesting the response to disease is not so very different [156,158]. More direct comparisons have been possible with the advent of *in vivo* ^31^P-MRS in living mice, which has shown that CK flux per gram of tissue is similar across species at ∼3 µmol/g and the reduction in this parameter observed in the failing heart is of a similar magnitude (∼50%) [159]. Ultimately, the evolutionary preservation of the CK system speaks to its importance across the mammalian species [160], yet we don’t observe exacerbation of heart failure when we deliberately impair it. So, while we can’t rule out species differences, nor is there an obvious smoking gun to explain it. It seems likely that these observations may reflect a high degree of physiological redundancy, with multiple layers of interacting systems to main ATP provision to an organ that is just too important to fail. Furthermore, as we will see in the next section, gain-of-function models have indicated that increasing CK activity is a potentially therapeutic strategy for heart failure and that work was also done in mice.

### Can an augmented CK system improve heart failure?

A more important question to ask is whether augmenting energetics can protect against heart failure. Drug treatment that reduces energy requirements may act to preserve PCr/ATP, but maximal creatine levels are tightly controlled in the heart, hence naturally occurring supraphysiological levels have not been described and creatine supplementation is ineffectual [19]. Note that this is in contrast to skeletal muscle, where creatine loading has been associated with mild improvements in muscle strength and endurance in patients with chronic heart failure [161,162]. Instead, the use of cardiac-specific mouse models over-expressing key components of the CK system have proved informative, albeit with mixed results (summarised in Table 2).

In a study of CrT-OE mice, ^1^H-MRS was used to pre-select myocardial creatine levels 20–100% above WT, with heart failure severity assessed six weeks after MI. As expected, creatine levels fell with the development of heart failure, but were successfully maintained at supraphysiological levels compared with WT hearts. Despite this, no improvements in mortality, LV structural remodelling or in function were observed [55]. A computer simulation based on data from a canine hypertrophy model predicted this result. It suggested that the reduction of total creatine in heart failure may be an adaptive response to maintain│Δ*G*_ATP_│in the face of falling adenine nucleotides, at least until a tipping point is reached. It further predicted that only a simultaneous increase in total creatine, total adenine nucleotides, and total exchangeable phosphates would improve │Δ*G*_ATP_│ in the failing heart [163]. We attempted to test this theory by feeding ribose to CrT-OE mice in order to overcome a bottleneck in purine *de novo* biosynthesis. This was successful in doubling the production of ribose-5-phosphate, but not in preserving TAN pool or improving LV remodelling and function [164]. Clearly other strategies are required to preserve TAN pool in the failing heart and test this hypothesis. However, in a small feasibility study, patients with coronary artery disease and heart failure were given oral ribose for 3 weeks, which improved diastolic function and quality of life scores [165].

The Weiss group performed a particularly elegant study using inducible MCK-OE mice [166]. When the transgene was switched on, PCr and ATP levels were unaltered, but total CK activity and CK flux increased by ∼70%. When these mice were subjected to pressure-overload and heart failure was assessed 8 weeks later, they had improved contractile function and survival, which was associated with a relatively preserved PCr/ATP ratio and higher CK flux. To demonstrate cause and effect the transgene was switched off in a subset of animals and the beneficial effect on function was subsequently lost [166]. Although unreported in the original paper, a later study notes that MCK-OE does not modify LV hypertrophy and dilatation in the pressure-overload model [61]. Nevertheless, this represents the first proof-of-principle evidence that augmentation of the CK system can have beneficial effects on function in the chronically failing heart. The authors’ state that this is causal evidence for energy starvation in the failing heart, which is true up to a point, but this is to ignore the flip-side, i.e. that the MCK-KO mouse does not exhibit obvious LV dysfunction [167,168].

A similar study has been reported in MtCK-OE mice subjected to pressure overload with follow-up at 6 weeks. Comparing sham-operated control hearts, the total CK activity was 37% higher in the overexpressing animals, while PCr/ATP was unchanged. Both parameters were reduced with pressure-overload, but successfully maintained above healthy control levels in the MtCK-OE hearts. However, this did not translate into attenuation of LV remodelling or improvements in cardiac function, even under maximal stimulation, although there was a small trend for reduced early mortality [169]. Thus, there was a complete disconnect between energetic and functional parameters.

The Wiess lab have recently reported on their own inducible MtCK-OE mouse [61], which had normal PCr and ATP levels, but higher CK rate constant and CK flux in the sham-operated animals. Ten weeks after surgery to induce pressure-overload, ATP levels in the MtCK-OE mouse were better preserved and there was a much smaller reduction in CK reaction velocity and flux. This was accompanied by more favourable LV remodelling, including reduced LV hypertrophy and dilatation and a higher ejection fraction, although survival went unreported. These findings were directly related to CK enzyme activity since crossing these mice with GAMT-KO negated the positive effects on LV remodelling. This was further attributed to an improvement in mitochondrial function and reduced mitochondrial ROS, alongside higher expression levels of antioxidants [61]. This fits with the concept of MtCK directly controlling mitochondrial respiration by regulating ADP generation [170]. The main difference between the two MtCK-OE models appears to be one of gene dosage, with the latter study showing a much more robust overexpression of MtCK protein at 3-fold WT levels as opposed to only 1.5-fold [61].

The above studies provide proof-of-principle that increasing either M-CK or Mt-CK can improve cardiac function via energetic mechanisms in pressure-overload heart failure, in particular, Mt-CK which also ameliorated LV remodelling. It remains to be seen whether these positive effects would be additive to standard heart failure therapy or be effective in other aetiologies. Again, much will depend on the development of pharmacological tools that can augment CK activity.

## Summary and conclusions

The CK system clearly plays a central role in myocardial energy provision acting to maintain stable ATP levels in the face of fluctuating demand. In the setting of ischaemia, all the available evidence agrees that an intact CK system is essential to minimise injury and improve functional recovery and that augmentation of either creatine levels (via activation of the CrT) or CK activity is cardioprotective in animal models of I/R.

It is a universal finding in chronic heart failure that the CK system is down-regulated, both in term of creatine levels and CK activity, contributing to a reduced myocardial energy reserve, thereby limiting the contractile reserve. A substantial body of evidence has reported correlations between energetic parameters and LV remodelling, mechanical function and even survival, suggesting that impaired energetics contribute to pathophysiology and help drive the heart failure phenotype. However, it is an inconvenient truth that rodent models with much more drastic creatine or CK deficiency than naturally occurs in the failing heart have a remarkably mild cardiac phenotype and that these deficits do not exacerbate the development of surgically-induced heart failure. Nevertheless, increasing either Mt-CK or M-CK activity has been shown to ameliorate CHF in murine models. Ultimately, the question of causality can be put to one side and the focus moving forward needs to be how we can translate these positive findings into the clinic. For this, we need to identify novel pharmacological tools for the manipulation of creatine levels, CK activity and the preservation of adenine nucleotides.

## Data Availability

N/A; this is a review article.

## Open Access

Open access for this article was enabled by the participation of University of Oxford in an all-inclusive *Read & Publish* agreement with Portland Press and the Biochemical Society under a transformative agreement with JISC.

## Abbreviations

β-GPA: β-guanidinopropionic acid
5′-NT: 5′-nucleotidase
AGAT: arginine:glycine amidinotransferase
AK: adenylate kinase
CDS: creatine deficiency syndrome
CHF: chronic heart failure
CK: creatine kinase
Cr: creatine
CrT: creatine transporter (SLC6A8)
DCM: dilated cardiomyopathy
GAMT: guanidinoacetate *N*-methyltransferase
HCM: hypertrophic cardiomyopathy
HFpEF: heart failure with preserved ejection fraction
HFrEF: heart failure with reduced ejection fraction
I/R: ischaemia–reperfusion injury
KO: knockout
LV: left ventricular
LVH: left ventricular hypertrophy
M-CK: muscle isoform of creatine kinase
MI: myocardial infarction
mPTP: mitochondrial permeability transition pore
Mt-CK: mitochondrial creatine kinase
OE: overexpressing
OxPhos: oxidative phosphorylation
PCr: phosphocreatine
Pi: inorganic phosphate
ROS: reactive oxygen species
TAN: total adenine nucleotides
WT: wild-type
|ΔG_ATP_|: Gibbs free energy for ATP hydrolysis
